# Risk factors for non-communicable diseases in rural Uganda: a pilot surveillance project among diabetes patients at a referral hospital clinic

**Published:** 2011-11-29

**Authors:** Olivia Namusisi, Juliet N Sekandi, Simon Kasasa, Peter Wasswa, Nicholas T Kamara, Medard Bitekyerezo, Placid Mihayo, Sheba N Gitta, David Mukanga

**Affiliations:** 1 African Field Epidemiology Network (AFENET), Kampala, Uganda; 2 Makerere University College of Health Sciences- School of Public Health, Makerere, Uganda; 3 University of Georgia, College of Public Health, Atlanta, Georgia, USA; 4 Mbarara Regional Referral Hospital, Uganda

**Keywords:** Diabetes, surveillance, non-communicable diseases, Uganda

## Abstract

**Introduction:**

Despite an increasing recognition of non- communicable diseases (NCDs) in sub-Saharan Africa, there is lack of well established surveillance systems for these diseases. In an effort to understand burden of NCDs in low-resource settings, the African Field Epidemiology Network launched a pilot project in 2009 to routinely capture patient data in the diabetes clinic of Mbarara Regional Referral Hospital. The objective of this study was to determine the prevalence and, the gender- and age- specific distributions of common NCD risk factors among diabetic patients attending a referral hospital in rural Uganda.

**Methods:**

A relational Access database was designed to collect information on NCD risk factors. These included smoking, alcohol use, family history of diabetes, hypertension and body mass index. Univariate analyses were done and differences in proportions tested using chi-square P-values in STATA version 10.0.

**Results:**

A total of 1,383 patient records were analyzed, with 61% being female and mean age of 39.6 years (SD 15.8). About 24% had a family history of diabetes. Smoking and alcohol use were more prevalent among males (16.6% vs. 8.3%; p<0.0001) and (30.7 vs. 13%; p<0.0001) respectively. Overweight, obesity and hypertension were more prevalent in women (18.6% vs. 9.7%, 8.6% vs. 2.6%; p<0.0001, and 40.3% vs. 33%, p=0.018) respectively.

**Conclusion:**

This pilot project shows that use of hospital-based data is a valuable initial step in setting up surveillance systems for NCDs in Uganda. Risk factors for NCDs were both age and gender-specific and predominantly related to lifestyle. This suggests the need to design gender-sensitive prevention interventions that target lifestyle modification in this setting.

## Introduction

Non-communicable diseases (NCDs) have become a major public health concern both in developed and developing countries. NCDs were responsible for nearly half of the burden of diseases measured in disability-adjusted life years (DALYs) in 2005, both worldwide and in low/middle-income countries [1]. Non-communicable diseases (NCDs), including diabetes, cardiovascular diseases, cancers, and chronic respiratory diseases represent 46% of the global burden of disease and cause an estimated 35 million deaths each year [2]. An estimated 80% of NCD deaths occur in low and middle income countries [3]. Moreover, WHO projects that NCDs death will increase by 17% over the next ten years. The greatest increase will be seen in the African region and the Eastern Mediterranean region [4].

In Sub-Saharan Africa, much attention has been paid to communicable diseases yet there is evidence to suggest that the burden of NCDs is increasing rapidly in most parts of the region [5–6]. Here, the prevalence of NCDs is projected to cause nearly 75% as many deaths as communicable, maternal, perinatal, and nutritional diseases by 2020 [2]. Among the most common NCDs, diabetes has emerged as a leading problem second to cardiovascular diseases. According to International Diabetes Federation (IDF) Diabetes Atlas, the global diabetes prevalence in the age group 20–79 years is estimated to be 6.6% for the year 2010 which translates into 285 million people suffering from the disease [7]. The rising prevalence of diabetes in the region has largely been ascribed to changes in lifestyle and urbanization, resulting in greater levels of obesity and physical inactivity [8].

NCDs are mainly associated with four shared behavioural risk factors including tobacco use, unhealthy diets, insufficient physical activity and the harmful use of alcohol [4]. Up to 80% of heart disease, stroke, and adult onset diabetes could be prevented by eliminating these behavioral risk factors. In Uganda, studies have documented hypertension, obesity and being overweight as common risk factors for diabetes [9–11]. Family history of diabetes, increasing age and ethnicity have also been associated with type 2 ( adult onset) diabetes [7]. However, the relative contribution of putative risk factors is not well defined and further research is therefore needed [12].

Surveillance could play an important role in collecting, analyzing and evaluating diabetes and its risk factors in order to improve prevention, control and treatment services. Studies on the epidemiology of diabetes in sub-Saharan Africa are generally limited. For example, the 2010 International Diabetes Federation Atlas found that only four African countries possessed data on diabetes, most of which was over ten years old [7]. The WHO STEPwise approach to Surveillance (STEPS) is a simple, standardized method for collecting, analyzing and disseminating data in WHO member countries [13]. Adaptation and implementation of the WHO STEPwise approach by healthcare systems in developing countries will be an important first step in overcoming the data limitations [14].

In Uganda, a surveillance system for NCDs is yet to be established. There are few published studies that have focused on the prevalence of NCD risk factors, most of them have been done in rural populations where the risks are likely to be less prominent [9–11]. The African Field Epidemiology Network (AFENET), a networking and support alliance of field epidemiology training programs in Africa, in collaboration with the Mbarara Regional Referral Hospital (MRRH) established a pilot surveillance system for NCDs using data collected during patients’ visits to the diabetes clinic. The purpose of this pilot study was to determine the prevalence and, the gender- and age- specific distributions of common NCD risk factors among diabetic patients attending a referral hospital in rural Uganda.

## Methods

### Study Setting and Population

The pilot project was established at Mbarara regional referral hospital diabetes clinic. The hospital is located in Mbarara district found in western Uganda, with six neighboring districts in the hospital's catchment area. The district had an estimated population of 457,800 people in 2010 whose main socio-economic activity is subsistence farming [15]. The pilot project was launched to facilitate surveillance of diabetic patients in relation to their characteristics, major risk factors and management.

### Study Design

This pilot was a secondary data analysis using a cross-sectional design. Data for all patients who attended the diabetes clinic from January 2005 to March 2010 were captured in a relational database using Microsoft Access.

### Ethical Issues

Following preliminary consultative meetings with the Mbarara hospital administration and the diabetes clinic staff, permission to collect and use patient records was granted by the Mbarara hospital Executive Director and the doctor in charge of the clinic. The study protocol was also reviewed and approved by the Makerere University Higher Degrees Research and Ethics Committee, the institutional review board responsible for reviewing this study. A waiver of informed consent was also approved by the IRB after satisfying all the conditions required for this.

### Measurements

Variables collected at the diabetes clinic were patients’ age, sex, district of residence, age at first onset of diabetes and family history of diabetes. Blood pressure, weight, and height were measured objectively. Information on NCD risk factors (smoking, alcohol use and family history of diabetes mellitus were gathered by patient self-reports. Particularly, family history of diabetes was obtained by asking patients whether they knew of any family member who had been diagnosed or told they had diabetes. Body mass index (BMI) was calculated from the measured patient weight and height (weight (kg) / (height (m)^2^), and categorized into four levels based on standard WHO cut-offs; underweight < 18.5, Normal at 18.5-24.9, Overweight at 25-29.9 and Obese at ≥30. The variable hypertension was also created from the measured systolic and diastolic blood pressure and categorized into three categories according to WHO; Normal at systolic blood pressure (spb) <120mmHg and diastolic (dpb) at < 80mmHg; Pre-hypertensive at spb 120-139 and dpb at 80-89; Hypertensive at sbp=140 and dpb=90. Random blood sugar levels (RBS) were measured during the patients’ first visit. Patients whose RBS was >11.1 mmol/L were considered to be to have poor gylcaemic control.

### Data Management and Analysis

Baseline data obtained from the patients’ records following their initial visit and new data captured during subsequent visits to the diabetes clinic was entered into an Access database designed by an expert biostatistician. All the old patients’ records from January 2005 to March 2007 were entered into the database. A data sheet was then completed on each visit by the clinicians using a simple one page tool which was placed in the patients clinic file. A data clerk extracted the information and entered it in the relational database 2-3 days following each patient visit. All patient records were manually entered by a trained data clerk and analyzed using STATA version 10.0 for descriptive statistics. Univariate analysis was to generate proportions and chi-square P-values to test difference in proportions for categorical variables.

## Results

### Participant Baseline Characteristics

A total of 1383 patient records were captured. The majority of patients (61%) attending the Mbarara referral diabetes clinic were female and the average age at first visit was 39.6 years, SD (15.8). Of the total number, 2.2% were below 18 years while nearly half of them (47.5%) were aged between 40 and 59 years. Nearly 24% of patients had a positive family history of diabetes. Six out of ten patients (60.7%) attending the diabetes clinic resided in districts neigbouring Mbarara whose distances range from about 30 to 90 km. Baseline details are shown in Table 1.

**Table 1:** Characteristics of Patients Attending the Mbarara Referral Hospital Diabetes Clinic, 2005– 2010

Characteristics	Frequency (n)	Percent (%)
**Sex**		
Female	844	61.0
Male	539	39.0
		
**Age in years†**		
< 18	30	2.2
18–39	265	19.2
40–59	657	47.5
60+	335	24.2
Missing	96	6.9
		
**Religion**		
Catholic	382	27.6
Protestant	992	42.8
Muslim	167	12.1
Others	31	2.2
Missing	211	15.3
		
Age at disease onset*		
<18 years	30	2.2
18–39	172	12.4
40–59	381	27.6
60+	119	8.6
Missing	681	49.2
		
**Family history of Diabetes**		
Yes	328	23.7
No	1,055	76.3
		
**District of origin**		
Mbarara	544	39.3
Isingiro	277	20.0
Bushenyi	200	14.5
Ntungamo	80	5.8
Kiruhura	60	4.3
Ibanda	32	2.3
Rukungiri	15	1.1
Others	175	12.7

† Mean age 39.6 yrs (SD= 15.8);

* Median age at disease onset 48 (IQR=38, 55.5) obtained by asking patients

**Figure 1:**
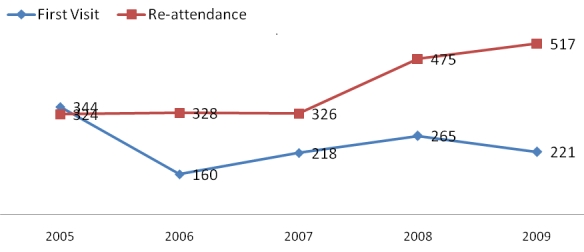
Number of new and re-attending patients of the Mbarara diabetes clinic by year

From 2005 to 2009, 1208 visits were recorded as first visits and 7239 as re-attendances. While re-attendance visits steadily increased from 2006 to 2009, the first visits fluctuated between 160 and 344 patients across the 5 years, with an average attendance of 242 new patients and 1456 re-attendances per year. Figure 1 shows the trend of the clinic visits during this period.

### Patients’ blood sugar levels on the first clinic visit

Random blood Sugar (RBS) was measured for 72.1% of the patients. About 64.1% had a RBS reading >11mmol/L while the mean RBS was 14.9 ± 7.5 mmol/L SD as shown in Table 2.

**Table 2:** Patients’ blood sugar level at first visit

RBS groups (mmol/L)	Patients	Percent
0–10	358	35.9
11 to 20	396	39.7
21 to 30	213	21.4
31 plus	30	3.00
Total	997	100

Mean ±SD: 14.9 ± 7.5 mmol /L; Median ±IQR: 13.0 ± 9.0

### Prevalence of Risk Factors for Non-communicable Diseases

Overall, the five commonly known risk factors; smoking, alcohol use, family history, abnormal weight and hypertension were prevalent among the diabetic patients attending the clinic. In total, 11.5% of the patients reported that they were smokers and 19.9% were alcohol-users. Among the reported risk factors, the prevalence of family history of diabetes ranked highest at approximately 24%, followed by alcohol use (20%) and smoking (11%). Many of the diabetic patients (44.7%) did not have their BMI defined because either weight or height measurements were missing. However, 42.4% had a normal BMI, while 9.5% were underweight and 6.3% were obese. Hypertension is also another risk factor associated with diabetes. The mean systolic blood pressure was 128.4 ± 12.5 and the mean diastolic blood pressure was 81.1 ± 13.0. Most of the diabetic patients were hypertensive, (37.5%) or pre-hypertensive (33.8%). Only 16.9% of the patients who had complete records were found to have normal blood pressure levels (Table 3).

**Table 3:** Distribution of major risk factors for non–communicable diseases by gender (N=1,383)

Characteristics	Male n = 537 (%)	Female n=846 (%)	Total	X^2^P–value
**Smoking**				
Yes	89 (16.6)	70 (8.3)	159 (11.5)	<0.0001
No	448 (83.4)	776 (91.7)	1,224 (88.5)	
				
**Alcohol Use**				
Yes	165 (30.7)	110 (13)	275(19.9)	<0.0001
No	372 (69.3)	736 (87)	1,108 (80.1)	
				
**Family History of Diabetes**				
Yes	110 (20.5)	218 (25.8)	328 (23.7)	0.024
No	427 (79.5)	628 (74.2)	1,055 (76.3)	
				
**Body Mass Index**				
Underweight	73 (13.6)	59 (7)	132 (9.5)	<0.0001
Normal	177 (33)	160 (18.9)	337 (24.4)	
Overweight	52 (9.7)	157 (18.6)	209 (15.1)	
Obese	14 (2.6)	73 (8.6)	87 (6.3)	
Missing	221 (41.2)	397 (46.9)	618 (44.7)	
				
**Hypertension**				
Normal	101 (18.8)	132 (15.6)	233 (16.9)	0.018
Pre–hypertensive	184 (34.3)	284 (33.6)	468 (33.8)	
Hypertensive	177 (33)	341 (40.3)	518 (37.5)	
Missing	75 (14)	89 (10.5)	164 (11.9)	

BMI= weight/ height ^2^ (Underweight< 18.5; Normal=18.5–24.9; Overweight= 25–29.9; Obese >=30); Hypertension (Normal= sbp<120; dpb<80mmHg; preHypetensive; spb120–139, dbp 80–89, Hypertensive; sbp >= 140, dbp >=90); Mean BMI = 23; Mean systolic blood pressure = 128 mmHg

### Distribution of Risk Factors for Non-communicable diseases by Gender

There were significant gender-specific differences in distribution of common risk factors for non-communicable diseases. Smoking was twice as prevalent in males compared to female patients (16.6% vs. 8.3%). Alcohol use was more than twice as prevalent among male patients compared to females (30% vs. 13%). In contrast, a slightly higher proportion of females reported a positive family history of diabetes (25.8% vs. 20.5%). Abnormal body mass index (overweight or obese) and hypertension were more prevalent risk factors among females compared to males attending the diabetes clinic as show in Table 3.

### Distribution of Risk factors for Non-communicable diseases by Age

There were significant differences in all NCD risk factors by age category but there were no distinct patterns in the prevalence of smoking, alcohol use, family history and BMI across age categories. However, age was an important risk factor in the study population. There was an increasing trend in proportions of patients with hypertension as age increased, ranging from about 22% to 48% (p<0.0001) among adults aged 18 years and older. Across all adult age categories, there was a slight but significant variation in self-reported smoking from approximately 10% to 14% (p=0.009), alcohol use also significantly varied from 17% to 23% (p=0.001), while positive family history varied from 21% to 27% (p<0.0001). The 40-59 year age group had the highest proportion (18%) of overweight cases compared to all other age groups. Obesity featured less prominently in this study sample, with prevalence ranging from 5% to 8% across age groups as shown in Table 4.

**Table 4:** Distribution major risk factors for non-communicable diseases by age group

	Age groups in years					
		
Characteristics (N=1,383)	< 18 n = 30 (%)	18–39 n=265 (%)	40–59 n=657(%)	>60 n=337 (%)	Missing N=96	X^2^P- value
**Smoking**						
Yes	0	25(9.4)	86(13.1)	44(13.1)	4(4.2)	<0.0001
No	30(100)	240(90.6)	571(86.9)	291(89.9)	92(95.8)	
						
**Alcohol Use**						
Yes	1 (3.3)	57(21.5)	151(23.0)	58(17.3)	8(8.3)	0.001
No	29 (96.7)	208(78.5)	506(77.0)	277(82.7)	88(91.7)	
						
**Family History of Diabetes**						
Yes	2(6.7)	62(23.4)	187(28.5)	69(20.6)	8(8.3)	<0.001
No	28(93.3)	203(76.6)	470(71.5)	266(79.4)	88(91.7)	
						
**Body Mass Index (BMI)**						**<0.0001**
Underweight	8(26.7)	37(14.0)	50(7.6)	29(8.7)	8(8.4)	
Normal	7(23.3)	69(26.0)	161(24.5)	87(26.0)	13(13.5)	
Overweight	1(3.3)	33(12.5)	119(18.1)	52(15.5)	4(4.2)	
Obese	0(0.0)	13(4.9)	52(7.9)	20(6.0)	2(2.1)	
Missing	14(46.7)	113(42.6)	275(41.9)	147(43.9)	69(71.9)	
						
**Hypertension**						
Normal	10(33.3)	68(25.7)	103(15.7)	38(11.3)	14(14.6)	<0.001
Pre- hypertensive	4(13.3)	93(35.1)	226(34.4)	108(32.2)	37(38.5)	
Hypertensive	2(6.7)	55(20.8)	263(40.0)	166(49.6)	32(33.3)	
Missing	14(46.7)	49(18.5)	65(9.9)	23(6.9)	13(13.5)	

The majority of the patients (58.6%) were on oral diabetic treatment with tablets and only 3.2% of the patients were on combination treatment of Insulin with tablets. About one in every four patients was treated with insulin alone during their first visit as shown in Table 5.

**Table 5:** Treatment received by the diabetes patients during the first visit

Type of treatment	Number of patients	Percentage
Tablets	811	58.6
Insulin	349	25.2
Tablets and insulin	44	3.2
Not stated	179	12.9
Total	1,383	100.0

## Discussion

This pilot study is one of the first to document the baseline characteristics and prevalence of risk factors for Non-communicable diseases (NCDs) among diabetic patients attending a Regional Referral Hospital in rural Uganda. The findings from this project demonstrate initial steps to supporting the development, streamlining and strengthening surveillance systems for NCDs by the African Field Epidemiology Network (AFENET) in Uganda. Results also show that surveillance of NCDs is feasible in health care settings in a developing country and sets the stage for the STEPS survey recommended by the World Health Organization [13].

The patients attending the diabetes clinic were predominantly female; this gender-bias in clinic attendance could be an artifact resulting from differential use in healthcare between women and men. Previous studies have shown that females are better health seekers than males [16]. Conversely, the gender differences could be a true reflection of a higher prevalence of diabetes among women in the underlying population; however the available data is not sufficient to draw this inference. The majority of patients attending the diabetes clinic were middle aged adults, 40 to 59 years. This is consistent with age distributions observed in studies done in other developing countries [17] and is expected because age is a confounding risk factor for majority of non-communicable diseases including diabetes, cardiovascular diseases and cancers [18–19]. The slightly lower proportion of patients aged older than 60 years in this sample is not surprising because there is a relatively small population in this age bracket in Uganda [20].

Adult onset (Type 2) diabetes is primarily linked to lifestyle rather than genetic predisposition [7]. In our study most of the patients had diabetes onset between the age of 40 and 59 and did not have a family history of diabetes. This is consistent with what was observed in other studies done in Africa [17]. The emerging NCD epidemic we see could be due to an increase in intake of high- calorie diets, smoking, alcohol use and sedentary lifestyles secondary to the high economic development, increasing urbanization, marketing and industrialization in Sub-Saharan Africa[21–23]. Our findings highlight the 40-59 year old individuals as a potentially high risk- age group that could benefit from routine screening and other primary care prevention interventions [24].

Gender-specific differences in risk factors highlight the importance of risk factor surveillance and the need for targeted interventions. Smoking and alcohol use were predominantly higher among males and this is consistent with data from general population from Uganda Demographic and Health Survey of 2006 [20] and other developing countries [17]. Lifestyle modification of personal habits may therefore be valuable in mitigating the impact of NCDs especially among men. In our sample, women had a tendency to higher body mass index than men, with two-fold and nearly three-fold higher proportions of overweight and obese female patients respectively. This finding is consistent with the demographic health survey data for western Uganda. A study done in Uganda showed that obesity and female gender were highly correlated among diabetics [9]. Similarly, a multi-site study of risk factors for NCDs done in Tanzania, Rwanda and Malawi found that women were more obese than men [17]. However, the rates of obesity observed in urban settings have been shown to be higher than in rural settings with correspondingly high rates of NCDs [25]. Consistent with our findings, Maher et al (2011) found that hypertension was the most common risk factor associated with diabetes in a rural-based population in Uganda [11].

The percentage of female patients with hypertension as a co-morbidity was higher than for males. Taken together, the differences in these risk factors could possibly be explained by a greater tendency for women to be physically inactive compared to men [8,26–27]. BMI is greatly influenced by dietary practices like high intake of saturated fatty acids, tobacco use and low physical activity [8,28]. From a broader perspective, internationally established BMI cut-offs for overweight and obesity may need to be adapted to the African populations in order to be used as appropriate indicators for NCD risk [13]. This would enhance the value of the STEPwise when it is applied in African settings.

Age-specific differences in the prevalence of risk factors were significant. The observed increasing trend in prevalence of hypertension with increasing age is not surprising. Age has been documented elsewhere as an independent risk factor for hypertension [18]. The absence of distinct differences across age groups with regard to smoking, alcohol use, family history and abnormal BMI may suggest that the patients were homogeneous in their underlying profiles such as socioeconomic status such that age only contributed to their background risk for NCDs [27].

The findings of this secondary analysis should be interpreted in light of several limitations. The incompleteness of the patients’ records as shown by missing values for data on risk factors such as age, weight and height, blood pressure measurements limits our understanding of the full picture of diabetes in the study sample. This could be overcome in the future by setting up quality control checks during data collection and management in order to improve overall data quality. There is potential for referral bias due to the fact that these pilot data were collected at a regional referral hospital. The patients seen at this clinic are likely to be sicker making the findings less representative of the underlying population in Mbarara and the rest of the districts where the patients came from in Uganda. Hence, a population-based study should also be done to further understand the risk profile for NCDs in the general population that gives rise to the clinic patients. The lack of information about sources of referral also limited the ability to assess the effectiveness health care system such as quality of primary health care. The validity of the findings could have been compromised by social desirability especially due to self-reported smoking and alcohol use [29,30]. In the Ugandan context, this phenomenon may be more common among women due to socio-cultural factors.

Despite the limitations, these findings could be generalized to the Mbarara diabetes clinic patients and perhaps similar referral clinics in Uganda. Moreover, this pilot study is an important first step that demonstrates the value of disease and risk factor surveillance in Uganda This study contributes to the sparse published literature on non-communicable diseases in developing countries.

## Conclusion

This pilot project shows that a hospital-based data are valuable for setting up surveillance systems in Uganda. Risk factors for NCDs were both age and gender-specific and predominantly related to lifestyle. This suggests the need to design gender-sensitive prevention interventions that target the lifestyle modification in this setting. There is need to further strengthen surveillance for risk factors of NCDs and more research should be done to generate supportive evidence for appropriate interventions.
